# Chemerin facilitates intervertebral disc degeneration via TLR4 and CMKLR1 and activation of NF-kB signaling pathway

**DOI:** 10.18632/aging.103339

**Published:** 2020-06-11

**Authors:** Sunli Hu, Zhenxuan Shao, Chenxi Zhang, Liang Chen, Abdullah Al Mamun, Ning Zhao, Jinfeng Cai, Zhiling Lou, Xiangyang Wang, Jiaoxiang Chen

**Affiliations:** 1 Department of Orthopaedics, The Second Affiliated Hospital and Yuying Children's Hospital of Wenzhou Medical University, Wenzhou, Zhejiang Province, China; 2 Key Laboratory of Orthopaedics of Zhejiang Province, Wenzhou, Zhejiang Province, China; 3 The Second School of Medicine, Wenzhou Medical University, Wenzhou, Zhejiang Province, China; 4 The First School of Medicine, Wenzhou Medical University, Wenzhou, Zhejiang Province, China; 5 Molecular Pharmacology Research Center, School of Pharmaceutical Sciences, Wenzhou Medical University, Wenzhou, Zhejiang Province, China

**Keywords:** chemerin, senescence, intervertebral disc, nucleus pulposus, inflammation

## Abstract

Now days, obesity is a major risk factor for intervertebral disc degeneration (IDD). However, adipokine, such as chemerin is a novel cytokine, which is secreted by adipose tissue, and are thought to be played major roles in various degenerative diseases. Obese individuals are known to have high concentration of serum chemerin. Our purpose was to study whether chemerin acts as a biochemical relationship between obesity, and IDD. In this study, we found that the expression level of chemerin was significantly increased in the human degenerated nucleus pulposus (NP) tissues, and had higher level in the obese people than the normal people. Chemerin significantly increased the inflammatory mediator level, contributing to ECM degradation in nucleus pulposus cells (NPCs). Furthermore, chemerin overexpression aggravates the puncture-induced IVDD progression in rats, while knockdown CMKLR1 reverses IVDD progression. Chemerin activates the NF-kB signaling pathway via its receptors CMKLR1, and TLR4 to release inflammatory mediators, which cause matrix degradation, and cell aging. These findings generally provide novel evidence supporting the causative role of obesity in IDD, which is essentially important to literally develop novel preventative or generally therapeutic treatment in the disc degenerative disorders.

## INTRODUCTION

In the history of development economics, low back pain (LBP) has been regarded as a key factor of disability worldwide [1, 2]. People with LBP come from all ages and have a variety of basic diseases, such as metabolic diseases, diabetes, and obesity [3, 4]. Among many factors that may cause LBP [5]. However, intervertebral disc degeneration is considered to be the leading cause [6]. Moreover, Mechanical stress, inflammation, and natural aging are the possible influencing factors for disc degeneration [7, 8]. Of the all factors accounting for IDD, obesity, implicating as risk factors of mechanical stress, and inflammation in the pathological process of IDD [9]. Therefore, obesity may be a potential target for the treatment of IDD in near future.

In the past decades, the rapid development of obesity group has been observed in many westernized countries [10, 11]. Obesity has been also recognized as one of the most serious public health concerns worldwide. However, previous studies have reported that adipose tissue may play significant role in the degenerative joint diseases by secreting effective bioactive molecules called adipokines [12]. Since disc degeneration is similar to the pathological process of arthritis in disease, adipokine is associated with the occurrence of disc degeneration, which is consistent with previous studies [13].

Adipose tissue can secretes adipokine, which is a bioactive substance with many biological functions [14]. Adipokines can act on the local tissues, and arrive in the distance by circulation to play major roles in the pro-inflammatory or anti-inflammatory effects. The adipokine family which is known to us includes leptin, resistin, chemerin, adiponectin, and lipocalin 2. Most of them can induce inflammation. One of them, including adiponectincan can reduce inflammation, and has significant protective effects. However, adipokines have been reported to play major roles in the systemic diseases [15–17], including arthritis, and disc degeneration, known as the most common orthopedic disease [18–21]. According to data, leptin and resistin accelerated disc degeneration by inducing inflammation, and then substrate degradation [22–24]. No study has reported about the role of chemerin in intervertebral disc, so the objective of our study was to explore the relationship between chemerin, intervertebral disc, inflammation, and matrix degradation.

Chemerin, also called as tarzarotin induced gene 2 (TIG2) or retinoic acid receptor reactive protein 2 (RARRES2), secreted in 18 kDa precursor protein (Chem163S) form [25–27]. After post-translational C-terminal cleavage, precursor molecules convert to 16 kDa active molecule. The precursor protein has multiple enzymatic cutting sites, which can produce various subtypes under different proteases treatment, and has various biological functions [28, 29]. However, chemerin was firstly discovered by Nagpal, who identified a gene, which is regulated by tazarotin. Then many scientists and researchers discovered several chemerin receptors, such as G-coupled receptor chemokine-like receptor 1 (CMKLR1), G protein-coupled receptor 1 (GPR1), and C-C motif chemokine receptor like 2 (CCRL2). It has been reported that chemerin and its receptor are highly expressed in the white adipose tissue of rodents, and human specimens [30, 31]. Especially in the obese people, it is found that chemerin level is proportional to BMI [32], which means that compared with lean people. Obese people had higher chemerin level in serum, white adipose tissues, and other body organs. However, a recent study has shown that IL-1β, and TNF-α can induce chemerin synthesis [33].

The purpose of our study was to provide a comprehensive study on the relationship between chemerin and several inflammatory mediators, obesity, and intervertebral disc degeneration in the patients with obesity and related metabolic diseases. Our studies reveal that the role of chemerin, and its downstream molecules in the pathological process of disc degeneration, providing a potential therapeutic target in near future.

## RESULTS

### The expression of chemerin, and CMKLR1 levels in different degenerated human NP and blood tissues

**Figure 1:**
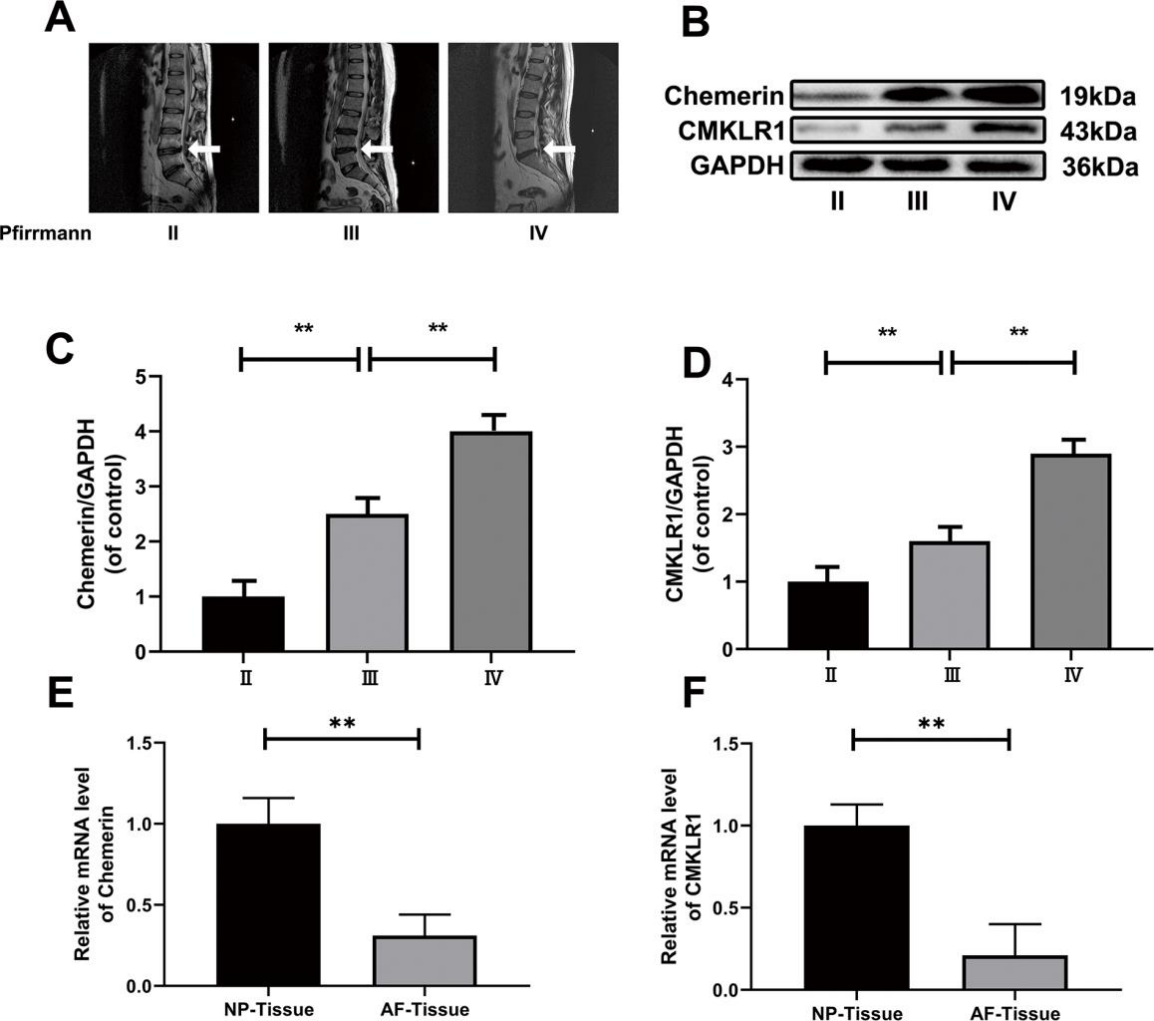
**The expression levels of chemerin, and CMKLR1 in different degenerated human NP and blood tissues.** (**A**) Representative MRI images of three different degrees of IDD patients. (**B**) The expression of levels of chemerin, and CMKLR1 from NPCs of different degrees of IDD patients were analyzed by western blotting. (**C**, **D**) Quantification of chemerin, and CMKLR1 immunoblots. (**E**, **F**) RT-PCR showed that the mRNA levels of chemerin, and CMKLR1 in the NP tissue were significantly higher compared with AF tissue. Data are represented as mean ± SEM of three independent experiments, each done in triplicate. Significant differences between groups are indicated as **p < 0.01, *p < 0.05.

In order to explore, the relationship between disc degeneration and chemerin, as well as CMKLR1, we performed western blotting to detect the NP tissue of patients with disc degeneration of different degrees. According to our results, chemerin and CMKLR1 levels in NP tissues were significantly increased with the increasing degeneration level (Figure 1A–1D). The expression levels of chemerin, and CMKLR1 mRNA in NP tissue were significantly higher compared to the expression levels in AF tissue (Figure 1E and 1F) (Table 1). Meanwhile, in order to confirm the relationship between obesity and chemerin, we collected the blood samples of asymptomatic volunteers of the same age group, and divided them into obese and normal groups. ELISA was used to measure the chemerin level, total cholesterol, triglycerides, HDL, and LDL (Table 2). The results showed that serum chemerin level in the obese group was higher than the normal group, and chemerin level was positively correlated with lipid metabolism (Table 3).

**Table 1:** Information of human disc samples from 10 patients.

**Human disc sample**	**Sex**	**Age (years)**	**Level**	**Diagnosis**	**Grade**
**1**	M	18	L2/3	L3 Fracture	II
**2**	M	20	L2/3	Disc herniation	II
**3**	F	21	T9/10	Disc herniation	II
**4**	F	19	L3/4	Disc herniation	II
**5**	M	28	L4/5	Disc herniation	III
**6**	F	31	L3/4	Disc herniation	III
**7**	M	34	T7/8	Disc herniation	III
**8**	F	46	L2/3	Disc herniation	IV
**9**	M	46	L4/5	Disc herniation	IV
**10**	F	46	L4/5	Disc herniation	IV

**Table 2:** Clinical features of asymptomatic volunteers.

**Variable**	**Control(n=50)**	**Obesity(n=50)**	***P* value**
**Sex**			.725
**Male**	28(56%)	27(54%)	
**Female**	22(44%)	23(46%)	
**BMI,kg/m^2^**	20.14±1.85	28.51±3.41	<.001^*^
**TC,mmol/L**	3.95±1.58	5.46±1.16	.002^*^
**TG,mmol/L**	1.61±1.21	2.42±1.03	.013^*^
**LDL-c,mmol/L**	2.20±0.91	2.98±0.65	.025^*^
**HDL-c,mmol/L**	1.31±0.16	1.06±0.13	.569
**Chemerin,ng/mL**	62.53±4.02	97.67±6.77	<.001^*^

Abbreviations: BMI, body mass index; HDL, high-density lipoprotein; LDL, low-density lipoprotein; TC, total cholesterol; TG, triglyceride.

*Indicates significant difference between groups (P < .05). P values derived from unpaired Student t test for normally distributed continuous variables

**Table 3:** Spearman rank correlations for selected clinical and laboratory makers with serum chemerin level in Asymptomatic population.

**Parameter**	**Spearman ρ**	**P value**
**Age(years)**	0.132	0.095
**BMI(kg/m^2^)**	0.296	0.000*
**TG(mmol/L)**	0.279	0.002*
**TC(mmol/L)**	0.036	0.019*
**HDL(mmol/L)**	-0.323	0.000*
**LDL(mmol/L)**	0.467	0.005*

Abbreviations: Age, maternal age at enrolment; HDL, high density lipoprotein; LDL, low density lipoprotein; BMI, body mass index; TC, total cholesterol; TG, triglycerides.

Values were obtained using Pearson’s correlation, and the other results were obtained using Spearman’s correlation. *Indicates significant difference between groups (P < .05).

### Effects of chemerin on the viability, and inflammatory responses of human NPCs

**Figure 2:**
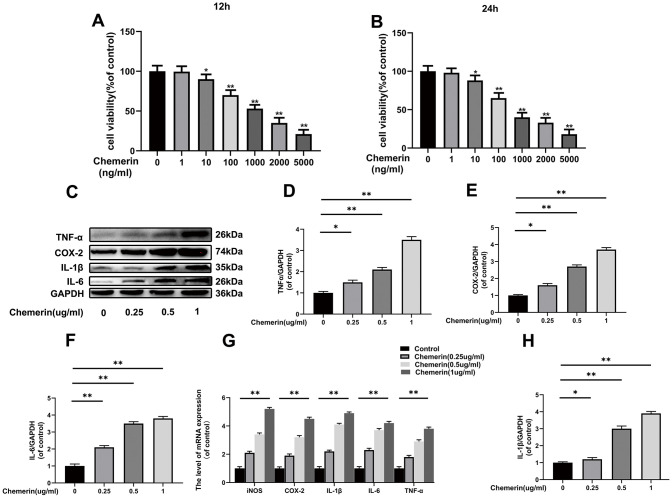
**Effects of chemerin on the viability, and inflammatory responses of human NPCs Human NPCs were treated with chemerin in a dose dependent for 12 or 24 hours.** (**A**, **B**) Cell viability of NPCs were determined by Cell Counting Kit-8 (CCK-8). (**C**) The expression levels of COX-2, TNF-α, IL-1β, and IL-6 were visualized by western blotting. (**D**–**F**, **H**) Quantification of COX-2, TNF-α, IL-1β, and IL-6 immunoblots in NPCs. (**G**) The mRNA expression levels of iNOS, COX-2, IL-1β, IL-6, and TNF-α in NPCs were evaluated by RT-PCR. Data are represented as mean ± SEM of three independent experiments, each done in triplicate. Significant differences between groups are indicated as **p < 0.01, *p < 0.05.

NPCs were cultured at different concentrations of chemerin (0, 1, 10, 100, 1000, 2000, and 5000 ng/ml) to measure the cytotoxic effect of chemerin using Cell Counting Kit-8 (CCK-8) for 12 or 24 hours. When the chemerin concentration was at 1*μ*g/ml, the cell activity of NPCs markedly decreased to about 50% for working 12 or 24 hours (Figure 2A, 2B). Therefore, the main experimental conditions were 1 *μ*g/ml, and 24 hours. In addition, we performed western blotting, and RT-PCR to confirm whether chemerin (0, 0.25, 0.5, and 1 *μ*g/ml) can induce inflammation. The western blotting results show that inflammatory mediators, such as IL-1β, IL-6, and TNF-α are markedly increased under chemerin stimulation. This result was also confirmed by RT-PCR. (Figure 2C–2H)

### Effect of chemerin on anabolism, and catabolism of ECM in human NPCs and AFCs

**Figure 3:**
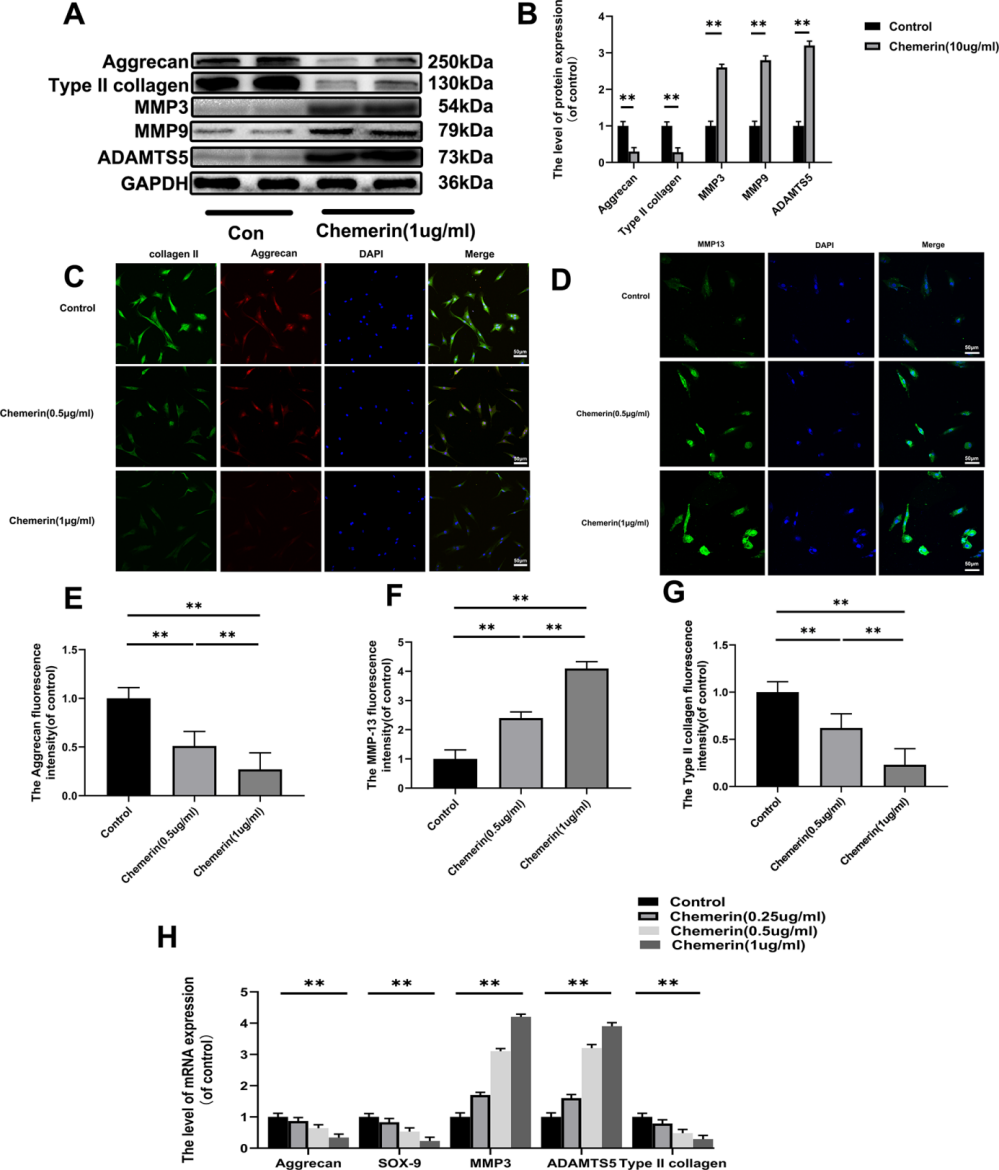
**Effect of chemerin on anabolism, and catabolism of ECM in human NPCs.** (**A**) The expression levels of aggrecan, collagen II, MMP3, MMP9, and ADAMTS5 were visualized by western blotting. (**B**) Quantification of Aggrecan, collagen II, MMP3, MMP9, and ADAMTS5 immunoblots. (**C**, **D**) The expression levels of collagen II, aggrecan, and MMP-13B were observed by immunofluorescence, and (**E**–**G**) the fluorescence intensity analyzed using Image J. (**H**) The mRNA expression levels of aggrecan, SOX-9, MMP3, ADAMTS5, and collagen II in NPCs were evaluated by RT-PCR. Data are represented as mean ± SEM of three independent experiments, each done in triplicate. Significant differences between groups are indicated as **p < 0.01, *p < 0.05.

Exploration the role of chemerin (1 *μ*g/ml, for 24 hours) in disrupting the ECM synthesis and degradation. The results of western blotting show that chemerin significantly reduce the expression levels of collagen II, aggrecan, and SOX9, which were important for the ECM synthesis, but promote the MMP9, MMP13, and ADAMTS5 production, which were regarded as main matrix degrading protein (Figure 3A, 3B). In addition, the results of cell immunofluorescence showed that collagen II, aggrecan, and MMP13 were in the nucleus and cytoplasm. After chemerin (0.5, and 1 *μ*g/ml for 12 hours) treatment, the abundance of collagen II, and aggrecan cell fluorescence were significantly decreased with increasing chemerin concentration (Figure 3C–3G). To further confirm the injury action of chemerin, the mRNA expression of related genes was assessed (Figure 3H). So, the results showed that chemerin promoted the expression levels of MMP3, ADAMTS5, and MMP9 but inhibited the expression levels of collagen II, SOX-9, and aggrecan. We also detected whether chemerin could significantly impact the AF cells. The results of immunofluorescence and western blotting showed that chemerin can influence matrix metabolism of AF, which is the same as NPCs (Supplementary Figure 1). In conclusion, the above results suggest that chemerin can significantly inhibit ECM synthesis, promote matrix degradation protein synthesis, and finally disrupt the balance of ECM synthesis and degradation.

### The AKT phosphorylation, and NF-kB signaling pathway were associated with chemerin induced cell damage.

**Figure 4:**
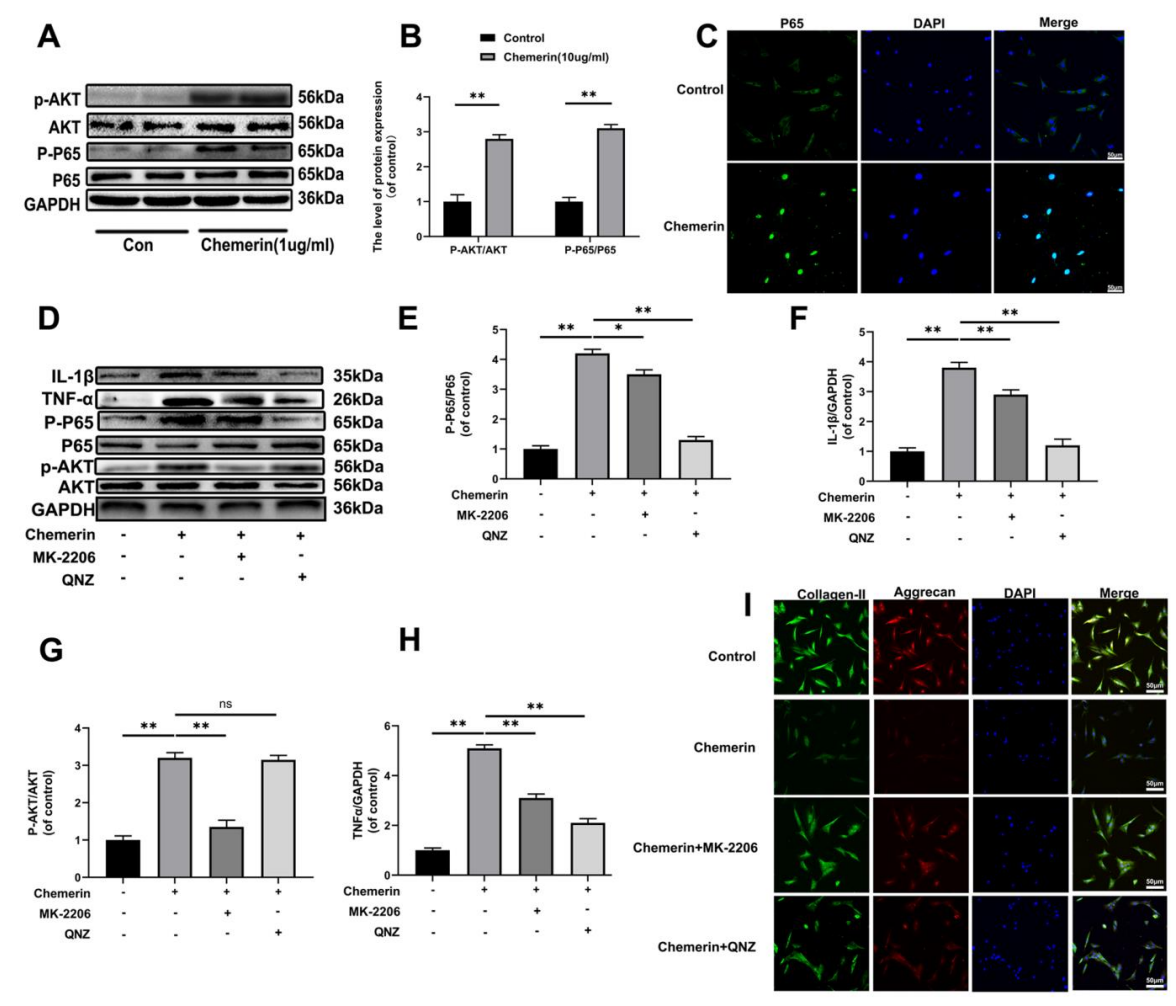
**The AKT phosphorylation, and NF-kB signaling pathway were associated with chemerin induced cell damage.** (**A**) The expression levels of p-AKT, AKT, p-p65, and p65 were visualized by western blotting. (**B**) Quantification of p-AKT, AKT, p-p65, and p65 immunoblots. (**C**) The expression levels of p65, and nuclear translocation were observed by immunofluorescence. (**D**) The expression levels of inflammatory mediators, and signaling pathway related proteins, such as IL-1β, TNF-α, p-AKT, AKT, p-p65, and p65 were analyzed by western blotting. (**E**–**H**) Quantification of IL-1β, TNF-α, p-AKT, AKT, p-p65, and p65 immunoblots. (**I**) Immunofluorescence of collagen II, and aggrecan in NPCs were observed by Nikon ECLIPSE Ti microscope (Nikon, Tokyo, Japan). Data are represented as mean ± SEM of three independent experiments, each done in triplicate. Significant differences between groups are indicated as **p < 0.01, *p < 0.05.

Several studies have demonstrated that inflammatory mediators play major roles in the progression of IVDD, and NF-kB pathway associated with aggravation of intervertebral disc degeneration. Thus, we performed western blotting to measure the inflammation-related proteins, such as p65, and p-p65 in NPCs under chemerin treatment (1 *μ*g/ml, 24 hours). In addition, studies have shown that AKT is one of the upstream molecules of NF-kB, and phosphorylation of AKT leads to the activation of NF-kB into the nucleus, triggering a series of inflammatory reactions. Our western blotting results showed that chemerin stimulation significantly increased the expression level of p-p65 and p-AKT without affecting the expression levels of total p65, and AKT (Figure 4A, 4B). Moreover, in this study, we also observed the translocation of p65 during chemerin induced NF-kB activation in NPCs according to the immunofluorescence staining of p65 (Figure 4C). The fluorescence intensity of p65 in nuclear significantly increased comparing to the control group. This phenomenon suggests that NF-kB signaling pathway was activated in chemerin stimulated NPCs to confirm the upstream, and downstream relationship between AKT and NF-kB in the chemerin-treated NPCs. We performed western blotting, and immunofluorescence using QNZ (10 μM) and MK-2206 (20μM), two kinds of pathway inhibitor target p65, and AKT, respectively. With the treatment of this inhibitor, the expression levels of p-p65, and p-AKT were markedly decreased. In addition, the use of QNZ did not affect the expression level of p-AKT, whereas MK2206 reduced the expression level of p-p65 (Figure 4D–4H). In addition, these inhibitors can alleviate chemerin-induced ECM degradation, which was shown in (Figure 4I). So, AKT and NF-kB pathways are associated with chemerin-induced NPCs damage.

### TLR4 and CMKLR1 were involved in chemerin-induced signaling pathway activation, ECM disorder and inflammatory responses

**Figure 5:**
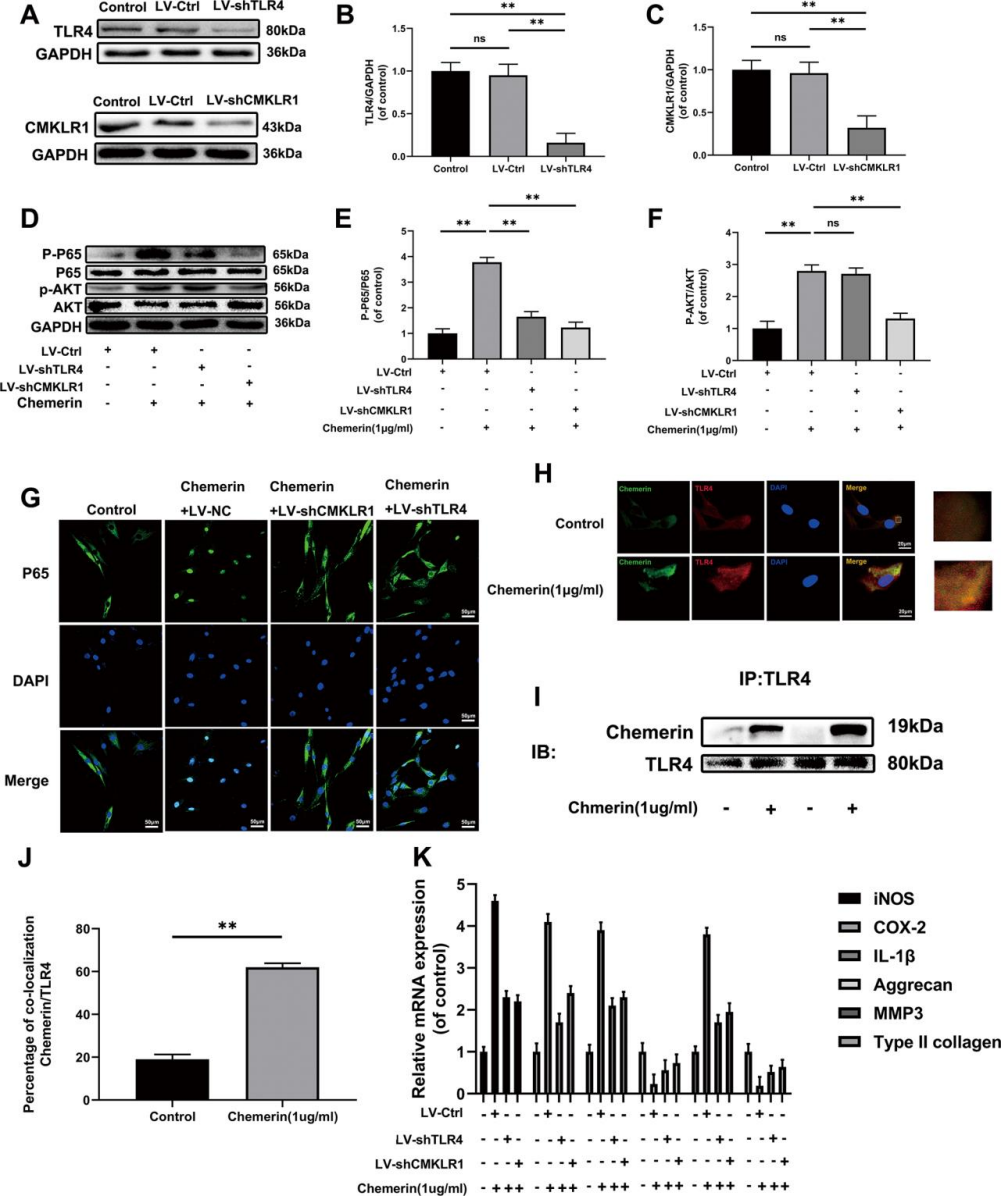
**TLR4, and CMKLR1 were involved in chemerin-induced signaling pathway activation, ECM disorder and inflammatory response.** (**A**) The expression levels of TLR4, and CMKLR1 were visualized by western blotting. (**B**, **C**) Quantification of TLR4, and CMKLR1 immunoblots. (**D**) The expression levels of p-AKT, AKT, p-p65, and p65 were evaluated by western blotting. (**E**, **F**) Quantification of p-AKT, AKT, p-p65, and p65 immunoblots. (**G**) The expression levels of p65, and nuclear translocation were observed by immunofluorescence. (**H**) Representative image of immunofluorescence double staining of TLR4, and chemerin in NPCs. (**J**) The quantification of the percentage of co-location of chemerin/TLR4 was detected by image J. (**I**) The co-immunoprecipitation data showed that compared with untreated group, the binding of chemerin to TLR4 was significantly increased after treatment with chemerin. (**K**) The mRNA expression levels of iNOS, COX-2, IL-1β, aggrecan, MMP3, and collagen II in NPCs were evaluated by RT- PCR. Data are represented as mean ± SEM of three independent experiments, each done in triplicate. Significant differences between groups are indicated as **p < 0.01, *p < 0.05.

To further explore the mechanism of chemerin inducing the activation of NF-kB pathway, TLR4 and CMKLR1 knockdown lenti-Virus were used to downregualte the protein expression. (Figure 5A–5C) showed that lentivirus reduced the expression levels of TLR4, and CMKLR1 precisely, indicating that there were no off-target effects. Activation of NF-kB signaling pathway induced by chemerin was reversed after using of Lv-shTLR4, and Lv-shCMKLR1 reduced AKT phosphorylation while Lv-shTLR4 did not, suggesting that TLR4 and CMKLR1 were in two different signaling pathways (Figure 5D–5F). Moreover, Lv-shTLR4, and Lv-shCMKLR1 could significantly suppressed the upregulated NF-kB nuclear translocation (Figure 5G). Besides, to confirm chemerin binding to TLR4, we performed co-ip, and immunofluorescence to co-location. Compared to the control group, chemerin binding to TLR4 was significantly increased by chemerin (1 μg/ml, 24 hours) treatment (Figure 5H–5J). The treatment of Lv-shTLR4 and Lv-shCMKLR1 markedly reversed the mRNA up-regulation of iNOS, COX-2, IL-1β and MMP3 also down-regulation of aggrecan, and collagen II induced by chemerin (1 *μ*g/ml, 6 hours) (Figure 5K).

### TLR4 and CMKLR1 knockdown attenuates the chemerin-induced senescence in human NPCs

**Figure 6:**
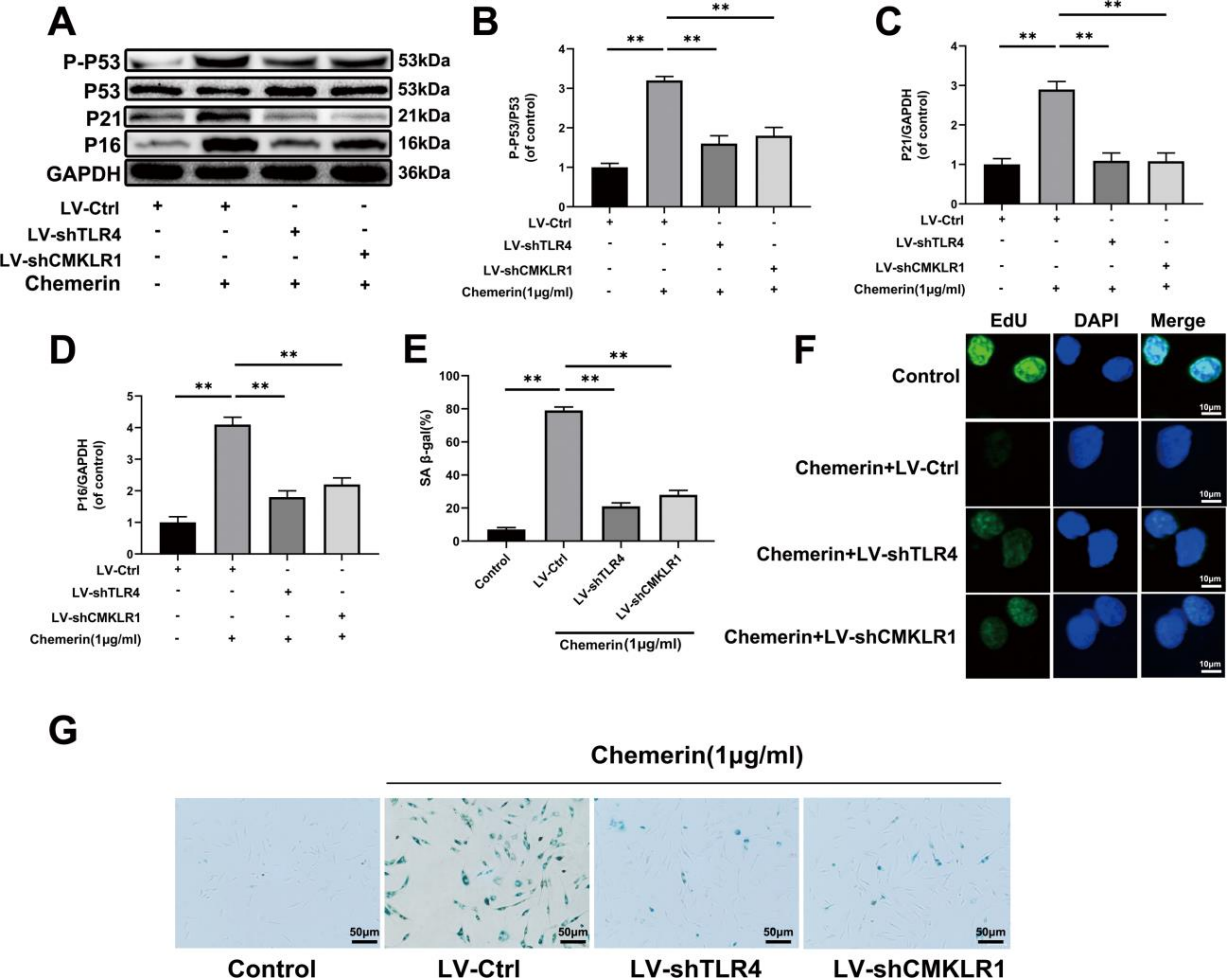
**TLR4, and CMKLR1 knockdown attenuates the chemerin-induced senescence in human NPCs.** (**A**) The expression levels of p-p53, p53, p21, and p16 were evaluated by western blotting. (**B**–**D**) Quantification of p-p53, p53, p21, and p16 immunoblots. (**E**, **G**) SA-β gal staining assay was performed in NPCs as treated above. (**F**) EdU microplate assay was performed in NPCs. Data are represented as mean ± SEM of three independent experiments, each done in triplicate. Significant differences between groups are indicated as **p < 0.01, *p < 0.05.

Aging is the main factor affecting the activity of NPCs. The protein expression of senescence-related makers, such as p53, and p16 were assessed by western blotting. The results show that p-p53, and p16 were markedly increased during chemerin stimulation, and reduced by Lv-shTLR4 and Lv-shCMKLR1 (Figure 6A–6D). In order to study whether chemerin promotes cell senescence, SA-β gal staining, a classical method was used to detect the cell senescence. Lv-shTLR4, and Lv-shCMKLR1 reduced the number of SA-β-gal positive cells in the chemerin-treated NPCs (Figure 6E, 6G). EdU assay, a method is widely used to detect the cell proliferation, which is decreased in senescent cell (Figure 6F). We found that chemerin could impair the proliferation capacity of NPCs, Lenti-shTLR4, and Lenti-shCMKLR1.

### Effects of chemerin aggravates IVDD through TLR4 and CMKLR1 in an ex-vivo model

To further investigate the effect of chemerin on IVDD, we constructed an ex-vivo animal model to verify the results of in-vitro experiments. In Supplementary Figure 2, administration of human recombinant chemerin markedly decreased the NP tissue volume. SO staining showed typical disc degeneration phenotype with the loss of NP cells and matrix. Nevertheless, Lv-shCMKLR1 and Lv-shTLR4 injection obviously delayed these histopathological changes, characterized by the existence of more NP cells.

### Chemerin aggravates the progression of IVDD in rat’s annulus needle puncture model partly through its receptor CMKLR1

**Figure 7:**
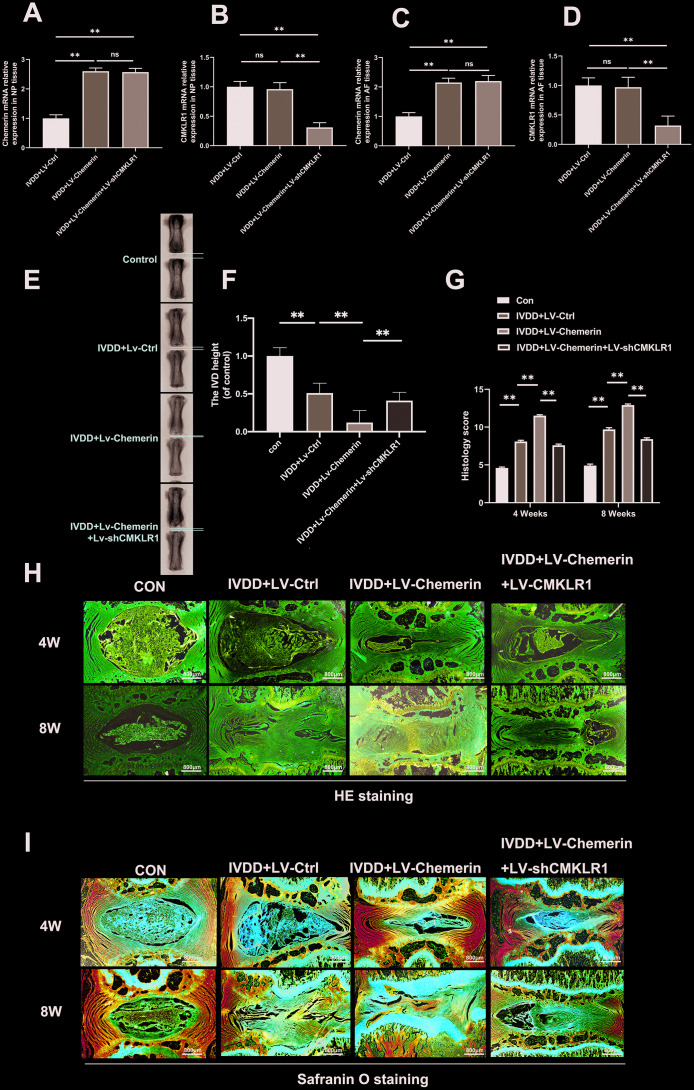
**Chemerin aggravates the progression of IVDD in rat’s annulus needle puncture model partly through its receptor CMKLR1.** (**A**–**D**) RT-PCR results showed that lentivirus-mediated target genes change in NPCs, and AF tissue were successful at 10 days after the lenti-virus injection. (**E**) X-ray of a rat tail with a needle-punctured disc at 8 weeks post-surgery. (**F**) The disc height index (DHI) was determined in four groups at 8 weeks. (**G**) The histopathological scores were evaluated at 4- and 8-weeks post-surgery in four groups. (**H**) Representative SO staining of punctured disc in different group (original magnification ×40, scale bar: 100 μm). Three sections were randomly selected for quantification, with a representative example shown. (**I**) Representative HE staining of punctured disc in different group (original magnification ×40, scale bar: 100 μm). Data are represented as mean ± SEM. Significant differences between groups are indicated as **p < 0.01, *p < 0.05.

In order to know the role of chemerin in the progression of IVDD in vivo model, we injected lentivirus into the rat intervertebral disc to overexpress chemerin, and inhibit CMKLR1 expression after IVDD surgery. We also performed X-ray, HE, and SO staining to estimate imageology and histomorphology changes. The chemerin mRNA levels in IVDD+LV-chemerin, and IVDD+LV-chemerin+LV-shCMKLR1 group were found to be increased in NP and AF tissues at 10 days after lentivirus injection. On the contrary, the CMKLR1 mRNA level was decreased in NP and AF tissues (Figure 7A–7D). From X-ray results, we found that the overexpression of chemerin accelerated the loss of disc height induced by the puncture surgery (IVDD surgery). However, downregulated expression of CMKLR1 alleviated the loss of disc height to some extent (Figure 7E, 7F). From HE and SO staining results in (Figure 7H, 7I) we could find that the gelatin NPCs gradually lost, and were replaced by the fibrous cells in the IVDD+ LV-NC group. The AF structures were lacerated or serpentine, then distended inward and even dislocated at 8 weeks after puncture, and the cartilaginous endplate was corroded and collapsed. The above changes observed in punctured discs were aggravated in the IVDD+ LV-chemerin group at 4 and 8 weeks. Nevertheless, LV-shCMKLR1 injection obviously delayed these histopathological changes. Histopathological scores from SO staining also showed that the harmful effects of chemerin, and its receptor CMKLR1 in IVDD progression (Figure 7G). These results demonstrate that chemerin plays significant roles in IVDD progression, and CMKLR1 may become a new potential therapeutic target in near future.

## DISCUSSION

In this study, we aimed to investigate the relationship between chemerin and inflammation. Therefore, we also confirmed the hypothesis that chemerin is associated with the occurrence of disc degeneration aggravated by obesity [34, 35]. We also confirmed for the first time the positive correlation between chemerin expression, and grade of disc degeneration. Although the association between obesity and disc degeneration has been elaborated in several studies, including basic experiments, and clinical cross-sectional studies, but there is no recognized mechanism to explain this phenomenon [35]. A large number of studies have demonstrated about the role of adipose tissue and its secreted products in metabolic diseases, and related diseases caused by metabolic abnormalities [36]. Thus adipose tissue is also considered as an endocrine organ [37]. So far, adipose tissue secretes more than 600 kinds of bioactive proteins. Among them, the potential role of adipokine has attracted more and more attentions in recent years, which can play various roles to regulate the energy expenditure, lipid metabolism, insulin sensitivity, glucose homeostasis, blood pressure, proinflammatory, and anti-inflammatory responses [38]. Adipokines are peptides that regulate the signal of functional status of adipose tissue to targets in the bone, gut, pancreas, brain, vascular system, and other tissues [39, 40]. Abnormal secretion of adipokine leads to the development of various diseases.

Chemerin is known as a adipokine family member, wihich has been shown to destroy cancer cells, and promote inflammation [41, 42]. However, some studies have indicated that chemerin can promote cancer metastasis, and inhibit inflammation [43]. Therefore, chemerin plays various roles in various systems. So far, no relevant study has described about the relationship between chemerin, and disc degeneration. Previous study has shown that chemerin can play significant roles in many musculoskeletal diseases, including osteoarthritis, and rheumatoid arthritis [44]. In addition, chemerin induces many inflammatory cytokines in arthritis. Similarly, intervertebral disc degeneration is also an inflammation-related disease, and the release of inflammatory factors plays an important role in the progression of disease. We wondered whether chemerin could induce inflammation in the intervertebral disc tissues.

We found that normal intervertebral discs expressed a small amount of chemerin, and its receptor, indicating that there was a certain role in the normal physiological functions. In several disc samples, the results showed that chemerin was induced during the disc degeneration. There are some resources of chemerin under the disc degeneration. Firstly, perivascular adipose tissue releases adipokines, which flows through the bloodstream to the vertebrae to function [45]. Secondly, chemerin is released by adipose tissue around the vertebrae as well as bone marrow fat, which affects the disc status. Study has demonstrated that vertebral marrow adipose tissue adipokine is a possible cause of intervertebral disc inflammation [46]. One report suggests that NPCs have cytokines, and chemokine receptors that bind with inflammatory mediators to induce intracellular inflammatory responses [47].

Our study is the first to demonstrate that chemerin activates the NF-kB signaling pathway through TLR4. TLR4 is a member of the TLR family, and expressed in most cells, mediating inflammation, stress, and injury [48]. Eisinger K et al. showed that in synovial fibroblasts, chemerin induces severe inflammatory responses through increasing the expression of TLR4, and the release of CCL2. [49]. Z.Liet al. showed that resistin activated the NF-kB and p38-MAPK signaling pathways through TLR4 receptor, resulting the up-regulation of CCL4 expression. These results suggest that TLR4 receptor plays major role in the inflammatory responses induced by adipokines. Therefore, we hypothesized that TLR4 is related to the inflammatory responses of NPCs induced by chemerin. Our data showed that TLR4 siRNA application could inhibit chemerin-induced activation of the NF-kB signaling pathway which was further confirmed with co-ip. The co-ip data showed that chemerin directly binds to TLR4 receptor, triggering the downstream reactions.

In disc degeneration, AKT is phosphorylated, and activated in NPCs, which was realized by the several inflammatory responses, including IL-1β, IL-6, and TNFα [50]. In our study, we found that the higher degree of AKT phosphorylation with increasing chemerin concentration. Furthermore, chemerin-induced AKT phosphorylation is independent of TLR4 up-regulation. After the use of siRNA TLR4, AKT phosphorylation induced by chemerin was not reversed. In order to study, the mechanism of AKT phosphorylation induced by chemerin, we focused on several chemerin receptors. Finally, we found that chemerin can markedly induce AKT phosphorylation by its receptor CMKLR1. However, CMKLR1 phosphorylated AKT in an indirect way, and the direct mechanism needs to be further studied. This result is consistent with previous study. AKT is one of the upstream molecules of NF-kB signaling pathway. AKT phosphorylation leads to the activation of NF-kB signaling pathway, and causes inflammatory responses [51].

**Figure 8:**
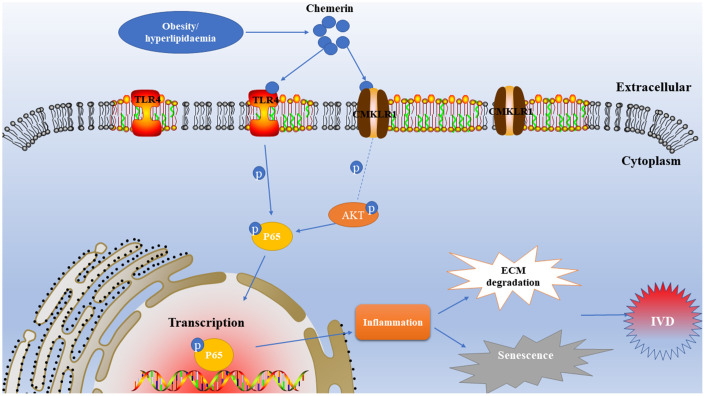
**Schematic illustration of the effects of chemerin in rats in the development of intervertebral disc degeneration.**

NF-kB plays major roles to regulate in the expression of IL-1β, IL-6, TNF-α, MMPs, and other inflammatory factors. The release of inflammatory cytokines and MMPs further damage the disc's structural proteins, such as proteoglycan, and collagen II. According to our results, chemerin activates the NF-kB signaling pathway through binding to the TLR4 receptor. In addition, chemerin binds to its own receptor CMKLR1 to phosphorylate AKT, and phosphorylated AKT further activates the NF-kb signaling pathway. In other words, chemerin activates the NF-kB signaling pathway together through two signal transduction pathways to trigger inflammatory responses (Figure 8). Prior studies have confirmed that chemerin plays significant roles in lipid, and glucose metabolism. To explore the effects of chemerin, and its receptor in IVDD model, we injected lentivirus into the rat’s intervertebral disc to knockdown the CMKLR1, and overexpress chemerin (Figure 7), then we performed X-ray, HE, and SO staining to estimate imageology and histomorphology changes. The results showed that the histomorphological, and immunological changes of rats injected with lentivirus to upregulate. Chemerin were worse than those treated with annulus needle puncture. However, these changes can be reversed to some extent by CMLKR1 knockdown. Hyperlipidemia, high total cholesterol, and high triglycerides are usually associated with high chemerin level, which is consistent with our experimental results.

Although there are some novel findings in our study, but some questions are remained to be further studied to clarify. Firstly, in order to improve chemerin level, and reduce to CMKLR1 level in NP tissues, we injected Lenti-virus with a microsyringe, but this was also a puncture injury to the intervertebral disc. Therefore, invitro experiment showed that chemerin could significantly accelerate the intervertebral disc degeneration process rather than chemerin inducing the intervertebral disc degeneration process. A non-invasive high level of blood or NP in chemerin animal model, and inhibitors of chemerin and its receptor inhibitors need to be studied in future. In addition, CMKLR1 activates the AKT signaling pathway in an indirect way. So, the actual mechanism of action needs to be further studied, whether chemerin activates the AKT signaling pathway in other ways.

Taken together, this study may explain the high prevalence of disc degeneration in obese patients. Moreover, these findings imply that chemerin, and its receptor may be potential therapeutic target for the treatment of obesity-related IVDD in near future.

## MATERIALS AND METHODS

### Ethical statement

The animal study was reviewed and approved by the animal experiments were conducted according to the Chinese law for the welfare of animal, and were approved by the Medical Ethics Committee of the Second Affiliated Hospital of Wenzhou Medical University.

### Human NP tissue, NPCs/ AFCs isolation and blood collection

Human NPCs and AFCs were isolated from different degenerated IVDs tissue respectively to compare chemerin level using western blotting [52, 53]. The patients were listed in Table 1. IVDs tissue was separated into NP and AF, which were cut up into 2-3 mm, and then washed for 3 times with phosphate-buffered saline (PBS) solution. After washing, tissues were digested with 0.25% collagenase II for 5 hours at 37 °C. Then the cells were washed by PBS for 3 times. After centrifuge, the cell suspension was cultured in the medium containing DMEM/F12 1: 1 (Invitrogen, CA, USA), 15% FBS (Invitrogen, CA, USA), and penicillin-streptomycin solution (100X) (Invitrogen, CA, USA). We collected blood sample from 100 asymptomatic volunteers and classified them as normal or obesity based on BMI whether more than 25 [54].

### NPCs/AFCs culture

NPCs and AFCs were extracted from patients who were listed in Table 1. The cells were cultured in the medium mentioned above. When the cell density reached more than 80%, cells were lifted and sub-cultured in three 10-cm dishes by 0.25% trypsin-EDTA (Invitrogen, CA, USA) solution in appropriate density. The medium was replaced every 3 days.

### Experimental design

In our study, in order to know the role of chemerin in inducing inflammation, cell senescence, and matrix degradation, different concentrations of chemerin (0, 0.5, 1μm/ml) were added in medium culturing for NPCs for 24 hours. To confirm that chemerin induces inflammation through the NF-kB and AKT signaling pathway, we used QNZ, an inhibitor suppressing effect of NF-kB mediated-inflammatory responses, and MK2260, an effective oral AKT allosteric inhibitor to perform the in vitro study.

### Lentivirus transfection

The cells were transfected with LV-shTLR4, LV-shCMKLR1 or LV- Ctrl ((Invitrogen, Carlsbad, CA, USA)) at a confluence of 40–50%; >95% of the cells were viable in 8 hours later. Then the medium was replaced with fresh medium in next 8 hours, the cells were incubated for a further 2 days, and passaged. Transfection efficacy was measured by western blotting.

### Western blotting assay

NPCs were lysed in RIPA buffer solution with 1 mM phenylmethanesulfonyl fluoride (Beyotime, China) at 4°C. BCA Protein Assay Kit (Beyotime, China) was used to assess the protein concentrations of sample. Protein samples of NPCs were separated on 10% or 12% SDS (sodium dodecyl sulfate) polyacrylamide gel electrophoresis, and were transferred to PVDF (polyvinylidene difluoride membrane) (Bio-Rad, Hercules, CA, USA) followed by blocking with 5% BSA (bovine serum albumin) in TBST (Tris-buffered saline with 0.1% Tween-20) for 2 hours. Then the membranes were washed by TBST for 3 times. After that, the membranes were incubated with specific primary antibodies to chemerin (1:1000), TLR4 (1:1000), TNF-α (1:1000), IL-6 (1:1,000), IL-1β, aggrecan (1:1000), collagen II (1:500), ADAMTS-5 (1:500), MMP-13 (1:1,000), AKT (1:1000), p-AKT (1:1000), p65 (1:1000), p-p65 (1:1000), CMKLR1 (1:1000), Histone H3 (1:1,000), and β-actin (1:1,000) at 4°C for overnight. In the next day, the membranes were washed by TBST for 3 times, and probed with secondary antibodies for 2 hours at room temperature. After reacting with the secondary antibodies, the signals were detected using a ChemiDoc XRS + system (Bio-Rad, Hercules, CA, USA). Finally, the results were quantified using Image Lab 3.0 Software (Bio-Rad, Hercules, CA).

### Biochemical tests

Total cholesterol (TC, mmol/L), high-density lipoprotein cholesterol (HDL, mmol/L), and triglyceride (TG, mmol/L) levels were determined for all subjects, using standard laboratory methods, and commercially available test kits (Roche Diagnostics GmbH, Mannheim, Germany). High-density lipoprotein (HDL, mmol/L), and low-density lipoprotein (LDL, mmol/L) values were obtained using the Friedewald formula.

### ELISA assay

Chemerin was measured in serum using a sandwich enzyme-linked immunosorbent assay (ELISA) (Human Chemerin DuoSet ELISA Kit, catalog No. DY2324, R&D Systems, Inc, Minneapolis, MN, USA) according to the manufacturer’s instructions.

### Cell proliferation assay

Proliferation ability of NPCs was determined by the Click-iT EdU microplate assay kit (Invitrogen) according to the manufacturer's instructions. Firstly, after the appeasement with different test compounds as described, NPCs were labelled with EdU miscible liquids. Then, EdU incorporated into DNA was detected using HRP-conjugated anti-Oregon Green antibody, and Amplex UltraRed. Finally, samples were observed in a confocal fluorescence microscope (Olympus Inc., Tokyo, Japan).

### Sa-β-gal staining

After washing with PBS solution for 5 minutes, cells on plates were fixed with 0.2% glutaraldehyde for 15 minutes at room temperature. Again, after washing with PBS for 3 times, cells were stained with X-gal staining solution at 37 °C for overnight. Finally, images were captured using Olympus IX71 microscope. NPCs showing blue represents a high degree of aging.

### Real-time PCR

TRIzol reagent (Invitrogen, Grand Island, NY) was used to extract the total RNAof NPCs. Then the total RNA was used to synthesize cDNA using machine (MBI Fermantas, Germany). For the RT-PCR, a total 10 μl of reaction volume was used for PCR amplification, including 5 μl of 2 × SYBR Master Mix, 0.5 μl of each primer, and 4 μl of diluted cDNA. Parameters of RT-PCR were: 10 minutes 95 °C, followed by 40 cycles of 15 seconds at 95 °C, and 1 minute at 60 °C. The cycle threshold (Ct) values were collected, and normalized to GAPDH level. The relative messenger RNA levels of each target gene were calculated by using the 2-ΔΔCt method. The primer sequences were listed in the following Table 4.

**Table 4:** Primer sequences used in qRT-PCR.

**Gene**	**Forward primer**	**Reverse primer**
**iNOS**	5’CCTTACGAGGCGAAGAAGGACAG-3′	5′CAGTTTGAGAGAGGAGGCTCCG3′
**COX-2**	5′GAGAGATGTATCCTCCCACAGTCA3′	5′-GACCAGGCACCAGACCAAAG-3′
**TNF-α**	5′-GTCAGATCATCTTCTCGAACC-3′	5′-CAGATAGATGGGCTCATACC-3′
**IL-6**	5′-GACAGCCACTCACCTCTTCA-3′	5′-TTCACCAGGCAAGTCTCCTC-3′
**IL-1β**	5′-TTCAAATCTCACAGCAGCAT-3′	5′-AGGTGGTCATCATCCCAC -3′
**Aggrecan**	5′-AATTTGAGAAGTCGTAATGC-3′	5′-AGGCCACTGTGCCCTTTTA-3′
**SOX-9**	5′-CTCCCAAAACAGACGTGCAA-3′	5′-CGAAGGTCTCGATGTTGGAGAT-3′
**MMP-3**	5′-GGTCCGATGTAACTCCTCTG -3′	5′-CCATGCTCCTTAATTCCAA -3′
**ADAMTS5**	5′-GTCTCAGCATTGACCTTCCGTG -3′	5′-ACAGGGAGTTCCATCTGCCACC -3′
**Type II collagen**	5′-CTCCCAAAACAGACGTGCAA-3′	5′-CGAAGGTCTCGATGTTGGAGAT -3′
**Chemerin**	5′-GGT CCA CTG CCC CAT AGA G-3′	5′-TTA TCA TGG CTG GGG ATA GAA-3′.
**CMKLR1**	5′-ACC TGC ATG GGA AAA TAT CCT-3′	5′-GAGGTTGAG TGT GTGGTAGGG-3′
**GAPDH**	5′-ACGGCAAGTTCAACGGCACA -3′	5GAAGACGCCAGTAGACTCCACGAC3′

### Immunofluorescence

The treated cells were firstly washed by PBS for 3 times fixed with 4% paraformaldehyde for 15 minutes. After washing PBS, the samples were treated with 0.5% (v/ v) Triton X-100 for 20 minutes and blocked by 5% BSA for 30 minutes at room temperature. Then, specific primary antibodies against collagen II (1:100), (1:200), aggrecan (1:50), MMP13 (1:100), p65 (1:100), TLR4 (1:100), and chemerin (1:50) were applied to the incubation of samples for overnight at 4° C. Then, the coverslips were incubated with specific Alexa Fluor®488-labelled or Alexa Fluor®594-conjugated secondary antibodies (1:200) for 1 hour at 37 °C, and stained with DAPI for 5 minutes. Finally, results were assessed in a confocal fluorescence microscope (Nikon, Japan). Image-Pro Plus image analysis system was used for the quantification of captured images.

### Ex-vivo IVDs from rats

Whole IVDs, including NP and AF were gently obtained from rats (male, 150–200 g, 8 week old), then left overnight before being randomized to three groups: (A) IVD; (B) IVD + chemerin; (C) IVD + chemerin + LV-TLR4; (D) IVD + chemerin + LV-CMKLR1 were given for 4 weeks after the photos were showed morphology changes, and safranin O fast green for proteoglycans and matrix degeneration. Caudal discs were isolated and cultured as previously described [55]. Caudal discs with complete endplates were isolated and cultured in DMEM containing 15% fetal bovine serum and 1% penicillin/streptomycin (Thermo Fisher Scientific, Waltham, MA).

### Surgical design

Adult male Sprague-Dawley rats (average weight 200–230 g) were purchased from the Animal Center of the Chinese Academy of Sciences in Shanghai, China housed in standard temperature conditions with 12-hours light/dark cycle, and regularly fed with food and water. IVDD rats were performed as described previously [56, 57]. During every puncture action, X-ray was used to check the location of needle and to make sure no endplate or bone injury. The SD rats were randomly divided into the four groups (n=12 in each):1-CON, 2-IVDD + LV-Ctrl, 3-IVDD + LV-chemerin, and 4-IVDD + LV-chemerin + LV-CMKLR1. After the rats were fully anesthetized by intraperitoneal injection of 2% (w/v) pentobarbital (40 mg/kg B.W), their tails and limbs were fixed with fixators. The experimental level of rat tail disc (Co3-4, Co5-6) was located by the digital palpation on the coccygeal vertebrae, and confirmed by counting the vertebrae from the sacral region in a trial radiograph. Needles (21G) were used to puncture the whole layer of AF though the tail skin [56, 58]. To make sure the needle won't be punctured too deep, the length of needle was decided according to the AF, and NP dimensions which were measured in the preliminary experiment is about 5 mm. All the needles were rotated at 360°, and kept in the disc for 1 minute. To eliminate the influences of the injected volume, only 5 μl lentivirus-control (LV-Ctrl) or lentivirus-chemerin (LV-chemerin) or lentivirus-shCMKLR1 (LV-shCMKLR1) were injected into the center space of the NP tissue through the inter-vertebral approach by using a microliter syringe with a needle of 27 gauge (10 μl, Gaoge, Shanghai, China). All operators were blinded to the animal experimental grouping.

### Histopathological analysis

Rats were sacrificed at 4 and 8 weeks after surgery the rats were executed using over-dosage of 4% pentobarbital, and the punctured segment (Co3-4, Co5-6) and non-punctured tails were harvested. The specimens were decalcified and fixed in formaldehyde, dehydrated, and embedded in paraffin. The tissue samples were cut into 5-μm sections. Slides of each disc were stained with Safranin O-fast green (SO), and haematoxylin and eosin (H.E). The cellularity and morphology of NP, and AF tissues were examined by a separate group of experienced histology researchers following blinded manner under microscope (Olympus Inc., Tokyo, Japan), and evaluated using a grading scale. The histolopathological scores were determined based on the histological appearance of the characteristics of NP, and AF tissues.

### X-ray image acquisition

After 8 weeks surgery, X-ray images were performed on all animals. After the administration of anesthesia by 10% pentobarbital (40 mg/kg) intraperitoneal injection, the rats were placed in the fairish position for X-ray image in a prone position for X-ray image by X-ray irradiation system (Kubtec). The disc height index (DHI) was measured using a previously described [59]. Percentage of disc height was calculated as the average of three measurements per disc.

### Co-immunoprecipitation (co-ip)

TLR4 co-ip was conducted using a commercial kit according to the manufacturer’s instructions (Pierce Crosslink IP kit). After 24 hours of chemerin treatment, NPCs were immediately placed on ice, and washed with ice-cold PBS solution for 3 times. Total protein was lysated as described above. To assess the chemerin-TLR4 binding, protein lysates were immunoprecipitated by incubating with Dynabeads containing TLR4 antibody at 4 °C for overnight. After overnight incubation, immunocomplexes were collected according to the manufacturer’s instructions. Whereas, the specific primary and secondary antibodies were incubated, and immunolabeling was detected by western blotting.

### Statistical analysis

The results were represented as mean±S.D. Statistical analyses were performed using SPSS (Chicago, Illinois, USA) statistical software program version 20.0. Data were analyzed by one-way analysis of variance (ANOVA) followed by Tukey’s test for comparison between control and treatment groups. Pearson or Spearman correlation analysis was used to analyze bivariate relations, and to test for associations between chemerin concentration and obesity measures, metabolic parameters, P < 0.05 was considered as significant value.

## Supplementary Material

Supplementary Figures
