# Supplementary material for: Chemerin facilitates intervertebral disc degeneration via TLR4 and CMKLR1 and activation of NF-kB signaling pathway
Source: Aging (Albany NY). 2020 Jun 11;12(12):11732–53. doi: 10.18632/aging.103339 (PMC7343479; doi:10.18632/aging.103339)
Supplement: Supplementary Figures [file aging-12-103339-s001..pdf]

SUPPLEMENTARY FIGURES

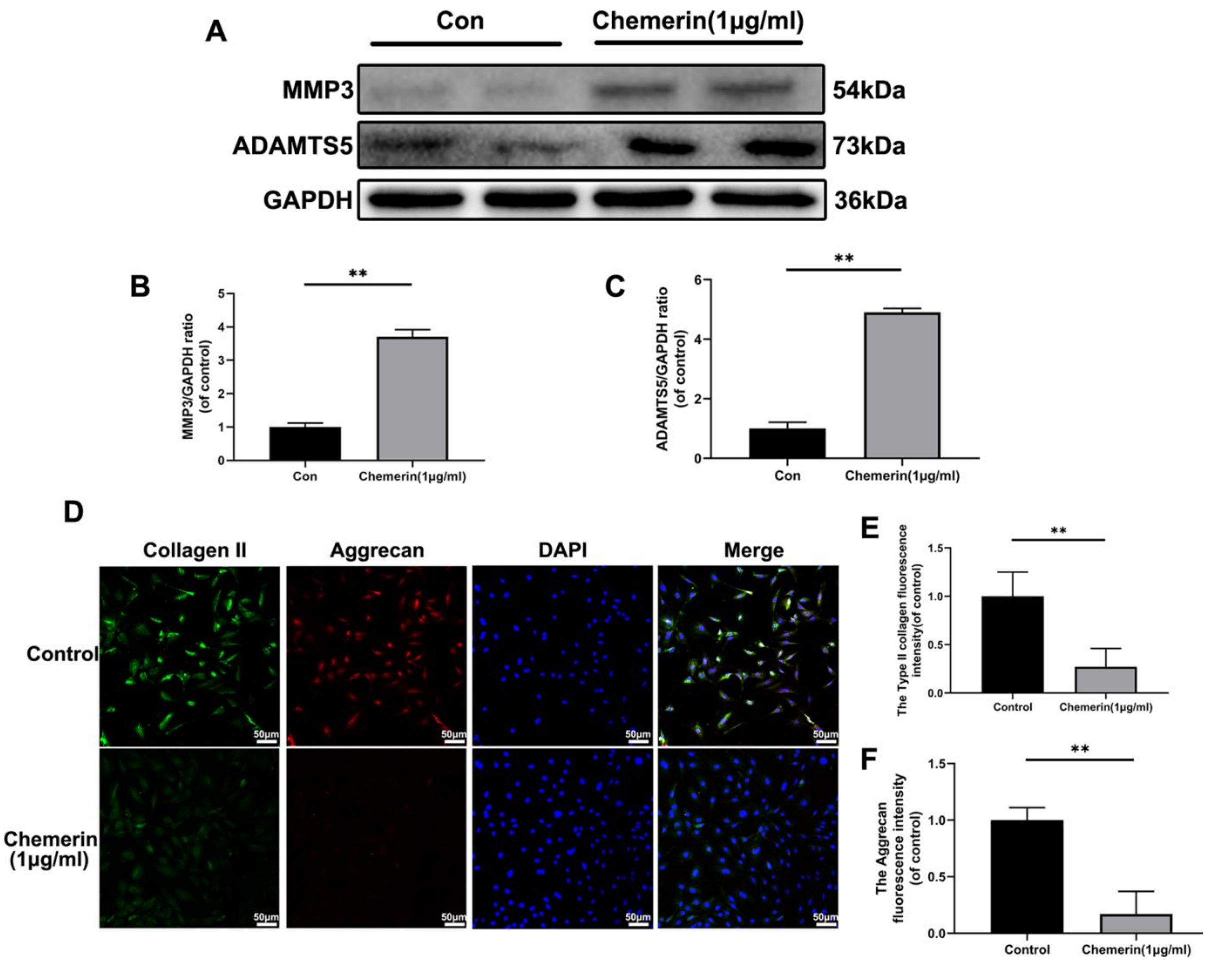

Supplementary Figure 1. Effect of chemerin on anabolism, and catabolism of ECM in human AFCs. (**A**) The expression levels of MMP3 and ADAMTS5 were visualized by western blotting. (**B**, **C**) Quantification of MMP3 and ADAMTS5immunoblots. (**D**) The expression levels of collagen II and aggrecan were observed by immunofluorescence, and (**E**, **F**) the fluorescence intensity analyzed using Image J. Data are represented as mean ± SEM of three independent experiments, each done in triplicate. Significant differences between groups are indicated as **p < 0.01, *p < 0.05.

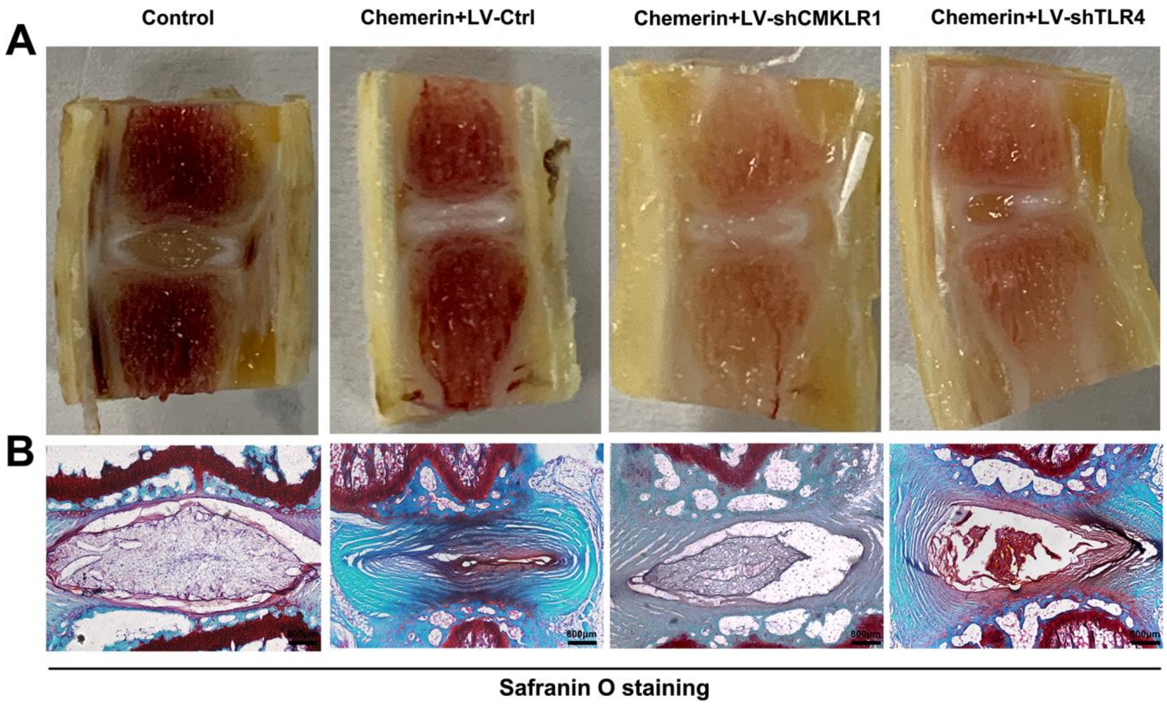

Supplementary Figure 2. Effects of Chemerin aggravates IVDD through TLR4 and CMKLR1 in an ex vivo model. (**A**) Digital photograph of Representative SO staining of punctured disc in different group. (**B**) Representative SO staining of punctured disc in different group.
